# Continuing Professional Development at Two Rural Hospitals in Ecuador

**DOI:** 10.5334/aogh.4175

**Published:** 2024-01-24

**Authors:** David Gaus, James Conway, Diego Herrera

**Affiliations:** 1 Andean Health & Development, US; 2 Ecuador, Brisas de Colorado, Sector 1, Via Quininde, Santo Domingo de las Tsachilas, Ecuador; 3 SMPH Office of Global Health, University of Wisconsin–Madison, School of Medicine & Public Health, 750 Highland Ave, Madison, WI 53726, US

**Keywords:** Continuing Professional Development, e-learning, hospital-based, Ecuador, Low Income Countries, Pedagogy

## Abstract

**Background:**

E-learning Continuing Professional Development (CPD) is an activity demonstrated to improve the quality of healthcare delivery. The CPD of medical and nursing staff in high income countries (HICs) is commonplace. CPD of administrative staff is less common, but increasingly frequent. In low- and middle-income countries (LMICs), CPD of any kind is infrequent, particularly in rural and remote areas.

**Objective:**

The aim of this study was to describe a hospital-based e-learning CPD program for clinical and non-clinical personnel as a unique example of a successful, ongoing educational pilot, quality improvement program involving a broad cohort of employees, in a country that does not require such activities.

**Methods:**

Using the online educational platform Chamilo, e-learning modules were created for eight groups including clinical and non-clinical employees. Upon completion of each module, one to two paragraph discussions were provided for each incorrect answer submitted. Two additional chances were offered for the employee to achieve a passing score of 70%. This study reports on the first 10-month period of the program.

**Findings:**

All participants achieved the 70% passing threshold after the first or second attempt. There was 100% participation by the employees required to complete the e-learning modules. Employee feedback suggested the modules were good for continuing education, but some felt the CPD was imposed on them.

**Conclusion:**

E-learning CPD is an important and emerging element for CPD and may provide opportunities for healthcare service quality improvement as part of broader pedagogical modalities, such as conferences and directed readings, in rural and remote areas of LMICs. These pilot programs could provide important information to develop Spanish-language e-learning CPD programs across a broader region, promote collaboration with regional professional societies, and possibly contribute to the establishment of national health program CPD standards.

## Introduction

The global shortage of healthcare workers poses a threat towards the accessibility of safe and appropriate care for the world’s population [1]. Another critical challenge is the availability of well-trained health staff [2]. Gaps in practice and knowledge can lead to poor outcomes. The implementation of *continuing professional development* (CPD) to maintain well-trained staff can contribute to improving quality of care. The combination of severely constrained financial resources, staff shortages, and insufficient healthcare infrastructure, along with gaps in knowledge and practice, can disproportionately affect LMICs [3].

In the international health sector, the term *continuing professional development* appears to be gradually replacing the term *continuing medical education*, suggesting the need for a wider range of competencies required for medical practice as well as the multidisciplinary nature of patient care [4]. The content of CPD is critical, as increasing academic knowledge alone should not be the primary goal of CPD. Rather, meaningful acquired knowledge occurs when CPD offers an opportunity for changes in practice [5].

The theory of adult learning, andragogy, suggests that adult learning styles are distinct from children and adolescents. Six principles in the theory of adult learning include the following:

- Adults are *results-oriented*: why is it important to learn something?
- Adults are *autonomous*: they want to control the techniques and goals within learning
- Adults have *life experiences and knowledge*: previous experiences have an impact on the learning process
- Adults are *relevancy-oriented*: there must be a motive to learn
- Adults are *practical*: they learn best when knowledge is presented in a real-life context
- Adults show a high *motivation to learn*: they seek information that can help them solve problems in their daily work [6].

Following successful application in various settings around the world, the andragogical model does not appear to be culture bound [7,8].

In North America, formal credit systems were established in 1948 to regulate the progression of medical education and was subsequently implemented in Europe [9]. In Ecuador, no process has been established to assess or certify professional competency, let alone in rural and remote areas [10]. Furthermore, medical and nursing professional societies as well as hospitals (or other healthcare facilities) do not require continuing medical or nursing education to maintain licensure or hospital affiliation.

Two of the authors have worked fulltime as clinicians and teaching faculty at two hospitals in rural Ecuador for 20 years, leading to the selection of these hospitals for this pilot intervention and study. These two hospitals have trained more than 70 family physicians who now work in a wide variety of settings in both the public and private sector throughout the country. Informal feedback from those graduates revealed that only very limited, intermittent, formal continuous professional development exists in the facilities where they are currently employed, such as occasional reviews of patient care at the facility. Some facilities inform their employees of opportunities for training and ongoing education offered by external organizations but do not offer any internal opportunities themselves.

The authors’ involvement in day-to-day operations of the two hospitals allowed for the identification of knowledge gaps across both clinical and administrative services, unique to the hospitals and to the rural context of patient care. While the CPD modules are based on these identified areas, they are not the only methods used for CPD. The two hospitals also use face-to-face meetings for CPD. The e-learning platforms were designed to complement face-to-face methodologies. Internet access is widely available in the surrounding community and is universally available within the hospitals.

Given this context, to improve quality of care, the authors created eight online CPD modules in Spanish-language for general medicine (residents working as part of a formal academic or non-academic program, and licensed family physicians), specialist physicians (some licensed family physicians also participated in these modules), nurses, pharmacy personnel, clinical laboratory personnel, environmental services personnel, administrative personnel directly involved in patient reception and billing, and nutrition-related personnel at two rural hospitals they manage. The organizational leadership agreed that continuing education (CE) for both clinical and non-clinical personnel was important and consistent with the concept of continuing professional development. This broader, multidisciplinary approach would potentially provide sustainable quality improvement for the institutions.

## Methods

The modules are all “question-based” with the exception of an occasional electrocardiogram for interpretation. No videos or other didactics were utilized. The initial module for physicians was composed of 30 questions, and feedback suggested that the module was too lengthy, with an average of approximately 1.5 hours to complete the module. Subsequently, it was reduced to a 10-question module. The online educational platform chosen was Chamilo, a free software e-learning and content management system. The authors created a 12-minute, Spanish-language, online video explaining how to enter and use this educational platform.

Chamilo offers a platform that allows for the creation of questions that, upon completing a given module, informs the learners if their answers are correct. Upon completion of the CE module, the learners are informed of their results and assigned a score. Scores above 70% are considered passing. Additionally, a one to two paragraph discussion of the answers and the reasons why they are correct or incorrect is provided for the learners. Having read the one to two paragraph discussion and learning theoretically why their answers were correct or not, learners are offered two more chances to pass the learning module.

Content for the general medicine (outpatient and hospitalist) modules include cross-cutting topics such as diabetes and hypertension, along with clinical cases (e.g., acute pancreatitis). Specialty physicians were also administered both cross-cutting topics and other focused questions pertinent to their specialty. Content for nursing modules was created by the chief of nursing, jointly with a nursing educator from the United States. Area supervisors created content for the other modules.

Pharmacy personnel are asked about how to prevent stock outs, how to present medications for nursing pickup, how to use the electronic medical record to assign medications to specific patients, and how to handle unused medications. Laboratory modules include questions about what specimen containers to use for specific blood samples and other body fluids, how to use the electronic medical record to determine what tests must be performed and how to register results. Content for the billing and reception modules includes, but is not limited to, questions around necessary documentation required from patients and referring institutions, and how to troubleshoot if that documentation is not presented at the time of admission. In the cleaning and maintenance modules, participants are asked about what types of detergents or disinfectants are used for various surfaces like flooring, patient beds, ventilators, and plastic tubing, to name a few. Food preparation and handling personnel are asked about the types of meals are provided to specific patient profiles, such as heart failure patients, diabetic patients, and postoperative patients. They are also asked about who is responsible for delivering trays to the hospital wards, what time they should be delivered and who is responsible for delivering trays to specific hospitalized patients.

The modules were introduced in February 2022 and have been completed monthly since that time. Some of the physician modules were performed twice monthly. Other areas have had only one or two modules since the project began.

Department leaders were responsible for promoting the upcoming evaluations and explaining the reasoning behind their administration and mandatory completion. Employees were also notified that the online evaluation must be completed before the end of each monthly pay period, or there would be a deduction from their salary. Employees were given sufficient opportunities during workdays to complete their online tasks.

## Results

Since February 2022, eight areas of e-learning evaluation have been implemented. Some areas have undergone more frequent evaluation than others. Table 1 summarizes the evaluations by department along with the number of participants, number of evaluations completed in a 10-month period, and distribution of the participants answering correctly at the 100%, 90% and 80% percentiles.

**Table 1:** Summary of Evaluations by Department.


DEPARTMENT	NO. PARTICIPANTS IN EACH DEPARTMENT	NO. EVALUATIONS OVER A 10-MONTH PERIOD	NO. PARTICIPANTS (%) WHO PASSED THE EVALUATION WITH A SCORE OF 100%	NO. PARTICIPANTS (%) WHO PASSED THE EVALUATION WITH A SCORE OF 90%	NO. PARTICIPANTS (%) WHO PASSED THE EVALUATION WITH A SCORE OF 80%

**General Medicine**	179	14	87 (49%)	53 (30%)	39 (21%)

**Specialist Physicians**	64	9	5 (8%)	2 (3%)	57 (89%)

**Nursing**	108	11	28 (26%)	53 (49%)	27 (25%)

**Pharmacy**	11	1	2 (18%)	5 (46%)	4 (36%)

**Clinical Laboratory**	68	1	25 (37%)	17 (25%)	26 (38%)

**Reception and Billing**	23	5	16 (70%)	2 (9%)	5 (21%)

**Food prep/handing**	17	2	7 (41%)	6 (35%)	4 (24%)

**Cleaning**	15	2	6 (40%)	2 (13%)	7 (47%)


The participants in the general medicine category include all medical residents in all areas of both hospitals. Some of these residents are part of an academic training program working towards a specialty, but most residents are not part of such an academic program. Participants in the nursing category include registered and auxiliary nurses. The clinical laboratory category includes laboratory staff personnel, but also nurses and administrative personnel involved in specimen handling and reporting. The food preparation and handling category also includes personnel from other areas involved in dietary logistics. The employment of new employees and the departure of others, particularly in the medicine and nursing categories, affected the number of participants.

All participants passed after the first or second attempt. No participants required a third attempt. All personnel (100%) that were designated to complete the e-learning modules actively participated. The Chamilo platform also allows for deeper analysis; the specialist physician group demonstrated that the average time taken to complete each module was seven minutes on the first attempt and four minutes on the second attempt.

## Discussion

The use of e-learning, or any educational intervention mediated electronically via web-based platforms, is increasing globally across all health professions. In practical terms, e-learning uses information technology to deliver education to remote learners. However, there is no standard definition of e-learning for research purposes.

The advantages of e-learning include lower costs, widespread distribution, increased access, frequent content updates, and personalized instruction, in terms of both content and pace [11]. Potential disadvantages include technology related costs, cost in program development, limited direct interaction, lack of interactions and relationship building between learners, decreased motivation to learn, requirement for greater self-discipline, as well as equity issues, such as poor access, language barriers, and lack of computer literacy [12].

In assessing the time requirements, the time taken to complete the 10 questions for physician modules was considerably less than what the educator team expected. One plausible explanation is that the learners determined very early in the process that they could answer all questions with any possible responses to gain immediate access to the correct answers. Subsequently, they would insert the correct answers without reading the comments section to understand what the correct answer was and why the other responses were incorrect. The educators elected to remove the correct answer from the response feedback section, forcing the learners to read the comments section, which contained the valuable learning opportunity to discover the correct answer.

The high uptake rate (100%) was not a surprising outcome, as participation was mandatory and tied to monthly salary payments. There was no difference in participation between the two hospitals. This result may have occurred due to the exceedingly aggressive approach, but the department leaders believed this to be the proper initial approach within an institutional culture of no previous CPD. The authors and department leaders hope to create an institutional culture of CPD where the participants appreciate the inherent value of CPD without having to tie that participation to salary. The authors believe this will take time and do not have a predetermined transition date established.

Initial feedback (achieved through informal discussion/convenience interviews with participants) from learners was mixed. Some physicians felt it was useful for continuing education. Others felt it was an imposition that was forced on them. One possible factor that led to this attitude was the disincentive imposed from hospital administration, requiring the modules to be completed; otherwise, employees faced a salary deduction at the end of each pay period. The educators are considering positive incentives such as a certificate of completion after a certain period (e.g., six months or one year).

Another challenge appeared to be the difficulty within the physician modules in finding cross-cutting themes for all physician specialties that include family medicine, adult critical care, neonatal critical care, general surgery and surgical subspecialties, and obstetrics/gynecology. To date, initial topics have included areas such as COVID-19, hypertension, and diabetes.

Ecuadorian culture does not have an avid reading culture, as the average Ecuadorian reads one complete book per year according to a national survey conducted by the Ministry of Culture and Patrimony [13]. Comparatively, in countries such as the United States, more than 12 books are read on average per year as reported by Gallop Poll in 2022 [14]. This limited reading habit in Ecuador could certainly impact interest in reading the questions, responses, and comments section in these modules.

The exact timeline and content have not been established to create a certificate of completion, but the hospitals expect to deliver certificates in certain areas (reception and billing, cleaning, food preparation, pharmacy) so that these employees can demonstrate knowledge acquisition when seeking future employment. Many persons in Ecuador seeking employment in these areas are hired as unskilled laborers, so these certificates can differentiate them from others. This was explained to employees in these departments, which likely contributed to their active participation.

The ultimate goal of this intervention is to improve patient care quality. While e-learning CPD modules have faced challenges to demonstrate this downstream impact in the literature, CPD does seem to help create a culture of ongoing learning and an expectation that any area in a hospital setting has an opportunity to implement better practices to improve patient care.

## Conclusion

E-learning is an important and emerging tool for continuing education that may provide opportunities for healthcare service quality improvement, in addition to other traditional pedagogical modalities such as conferences, in-service learning, and directed readings, among others. Creating the expectation that continuous educational activities are a workplace requirement could contribute to an overall environment of constant on-the-job learning across an organization and result in improvement of health care quality and client satisfaction. Few examples of continuing professional development via e-learning in Latin America exist in the medical literature, particularly in rural and remote areas.

The authors present one pilot program experience in rural Ecuador, with the intent to continue to modify and improve the program, as well as study program impacts on employee satisfaction and quality of health service delivery longitudinally. Examples of next steps include increasing the complexity of questions for medical trainees as they advance through their programs and establishing a pool of common core content material to emphasize key information for all personnel. Future institutional program opportunities include expanding access to graduates of the residency training programs. Ultimately, these programs could provide important information to develop Spanish-language CPD programs available across a broader region, promote collaboration with regional professional societies, and perhaps justify establishment of national health program CPD standards.
